# AGING SINGLE PARENTS: PSYCHOSOCIAL OUTCOMES AND WELL-BEING AMONG A MARGINALIZED POPULATION

**DOI:** 10.1093/geroni/igae098.2146

**Published:** 2024-12-31

**Authors:** Douglas Hanes

**Affiliations:** Stony Brook University School of Medicine, Stony Brook, New York, United States

## Abstract

Objective Over the last 50 years, single parents—in particular, single mothers—have received much popular and political attention. And yet, their aging experiences remain unresearched. This paper analyzes aging among single parents, including their sex/gender and racial/ethnic composition, as well as their psychosocial well-being, in a population-based aging study. Method Using Health and Retirement Study data (N = 38,785), we identify single parents as participants who were never married at baseline and have children (n = 680), 1.80% of participants with children. Multilevel regressions analyzed psychosocial outcomes: discrimination experiences, subjective social status, and social support. Results Odds of single parenthood increased for each subsequent birth cohort (OR = 1.89, p < 0.001). Women (□2 = 85.192, p < 0.001) and people of color (□2 = 802.32, p < 0.001) were more likely to be single parents, who also had lower education levels and wealth. Single parents were more likely to report discrimination due to race (OR: 1.522, p = 0.047), with single mothers of color more likely to report discrimination due to financial status (OR = 1.575, p = 0.049). Single parents received less positive social support and more negative support from their children; they were also more likely to experience depression, have lower self-rated health, and smaller social networks. Discussion Aging single parents are more likely to belong to already-marginalized groups, and face further and ongoing discrimination, known risk factors for adverse later-life health and well-being. These results indicate the need for greater attention to this marginalized group.

